# Building a community-academic partnership in public housing: Lessons from applying the CBPR Partnership Readiness Model

**DOI:** 10.64898/2026.10.28.26366741

**Published:** 2026-11-06

**Authors:** Nadia A. Sesay, Kendra OoNorasak, Jayden E. Brewer, Mansura Shahad Bawa, Dana Mason, Kristin Hughes, Jessica Coffie, Brandi M. White

## Abstract

The DASH (Dietary Approaches to Stop Hypertension) eating plan has been shown to reduce hypertension; however, adherence is low among low-income African American (AA) women, who experience a disproportionate burden of hypertension. To better understand barriers to DASH among low-income AA women, specifically AA women living in public housing, we established a community-academic partnership using a project advisory board (PAB). This paper applies the CBPR Partnership Readiness Model to assess community stakeholder readiness for the partnership. PAB members were recruited through ongoing research and volunteer work in the target community. We developed a 33-page PAB workbook clarifying study goals, member roles and expectations, and hypertension information. A questionnaire was used to gather feedback on members' experiences. PAB membership increased by 150% after the first year following a second recruitment phase. Members reported satisfaction with progress toward local food equity initiatives and with partnership sustainability. Findings indicate the model was helpful in evaluating the PAB formation process for a project involving public housing stakeholders. PABs can help build local support for community-engaged projects. When members can see the advantages of their involvement, partnerships are strengthened.

